# Robotic-Assisted Vascular Surgery: Current Landscape, Challenges, and Future Directions

**DOI:** 10.3390/jcm14207353

**Published:** 2025-10-17

**Authors:** Yaman Alsabbagh, Young Erben, Adeeb Jlilati, Joaquin Sarmiento, Christopher Jacobs, Enrique F. Elli, Houssam Farres

**Affiliations:** 1 Division of Vascular and Endovascular Surgery, Mayo Clinic, Jacksonville, FL 32224, USA; alsabbagh.yaman@mayo.edu (Y.A.);; 2 Department of Surgery, Mayo Clinic, Jacksonville, FL 32224, USA

**Keywords:** robotic surger, endovascular, AI, Da Vinci, aortic repair, nutcracker syndrome, MALS, TOS

## Abstract

Vascular surgery has evolved from durable yet invasive open reconstructions to less traumatic endovascular techniques. While endovascular repair reduces perioperative morbidity, it introduces durability challenges and the need for lifelong surveillance. Laparoscopic surgery bridged some gaps but was hindered by steep learning curves and technical limitations. Robotic-assisted surgery represents a “third revolution”, combining the durability of open repair with the recovery and ergonomic benefits of minimally invasive approaches through enhanced 3D visualization, wristed instrumentation, and tremor filtration. This review synthesizes current evidence on robotic applications in vascular surgery, including aortic, visceral, venous, and endovascular interventions. Feasibility of robotic vascular surgery has been demonstrated in over 1500 patients across aortic, visceral, venous, and decompression procedures. Reported outcomes include pooled conversion rates of ~5%, 30-day mortality of 1–3%, and long-term patency rates exceeding 90% in aortoiliac occlusive disease. Similarly favorable outcomes have been observed in AAA repair, visceral artery aneurysm repair, IVC reconstructions, renal vein transpositions, and minimally invasive decompression procedures such as median arcuate ligament and thoracic outlet syndromes. Endovascular robotics enhances catheter navigation precision and reduces operator radiation exposure by 85–95%, with multiple series demonstrating consistent benefit compared to manual techniques. Despite these advantages, adoption is limited by high costs, lack of dedicated vascular instruments, absent haptic feedback on most platforms, and the need for standardized training. Most available evidence is observational and from high-volume centers, highlighting the need for multicenter randomized trials. Future directions include AI-enabled planning and augmented-reality navigation, which are the most feasible near-term technologies since they rely largely on software integration with existing systems. Other advances such as microsurgical robotics, soft-robotic platforms, and telesurgery remain longer-term developments requiring new hardware and regulatory pathways. Overcoming barriers through collaborative innovation, structured training, and robust evidence generation is essential for robotics to become a new standard in vascular care.

## 1. Introduction

### 1.1. The Evolution of Vascular Surgery

Vascular surgery has historically been shaped by transformative technological innovations that have redefined treatment paradigms. For decades, open surgical reconstruction was the undisputed gold standard, offering superior long-term durability and patency compared with alternative approaches [1,2,3,4]. A large multicenter retrospective cohort study by Yei et al., utilized Medicare-matched Vascular Quality Initiative Vascular Implant Surveillance and Interventional Outcomes Network (VQI-VISION) database, demonstrated significantly lower six-year mortality after open abdominal aortic aneurysm (AAA) repair compared with endovascular approaches [5]. However, these benefits came at the cost of substantial operative trauma, increased perioperative morbidity, and prolonged recovery [6,7,8,9].

The advent of endovascular techniques in the 1990s, particularly endovascular aneurysm repair (EVAR), marked a second major paradigm shift [10]. These methods significantly reduced physiological stress, shortened recovery times, and expanded treatment eligibility to higher-risk patients. Despite these advantages, endovascular repairs introduced challenges, including higher rates of late rupture, secondary interventions, and the necessity for lifelong surveillance using computed tomographic angiography (CTA) or duplex ultrasound [11,12,13,14,15,16,17,18,19]. Repeated CTA imaging raises concerns regarding cumulative radiation exposure and nephrotoxic contrast use, particularly in younger patients.

In the same era, laparoscopic vascular surgery emerged as an intermediate step, aiming to merge the durability of open reconstruction with reduced surgical trauma. Totally laparoscopic and hand-assisted laparoscopic surgery (HALS) for AAA repair demonstrated benefits such as faster gastrointestinal recovery, improved wound healing, reduced incidence of incisional hernia, and better cosmetic outcomes [20,21]. Nonetheless, the steep learning curve, limited instrument mobility, and reliance on two-dimensional visualization hindered its widespread adoption, particularly for technically demanding vascular reconstructions [22].

### 1.2. The Dawn of the “Third Revolution”: Robotics

**Figure 1:**
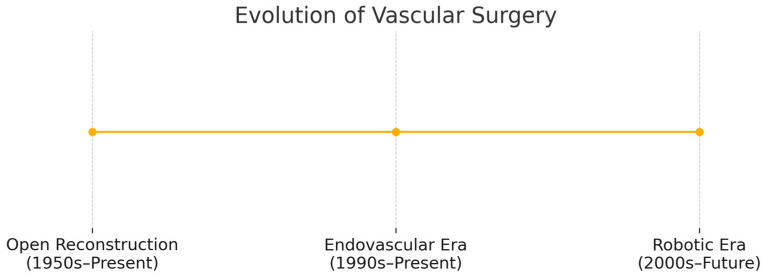
Evolution of Vascular Surgery: From Open Reconstruction to the Robotic Era. A graphical timeline illustrates the transition from open surgery to endovascular, laparoscopic, and robotic-assisted vascular surgery, highlighting key technological milestones and landmark studies.

Robotic-assisted surgery was introduced to overcome many of the ergonomic and technical limitations of laparoscopy. First reported for aortic surgery by Wisselink et al. in 2002 [23], robotics provides high-definition three-dimensional vision, articulated instruments with seven degrees of freedom, motion scaling, and tremor filtration. These features restore and often exceed the dexterity of the human hand, enabling fine dissection and precise suturing in confined anatomical spaces (Figure 1).

The appeal of robotics in vascular surgery lies in its potential to combine the durability of open repair with the minimal invasiveness of endovascular approaches, offering improved patient recovery without sacrificing long-term outcomes. However, adoption has been slow due to several hurdles. High costs remain a major barrier, with the capital investment for a robotic system approaching $1.5–2.0 million and annual service fees of approximately $100,000–180,000. Analyses suggest that a center must perform 100–120 robotic cases annually to achieve financial sustainability, a volume that many vascular programs, unlike other high-volume specialties, may be unable to reach. Additionally, the absence of dedicated vascular instruments is a challenge, as tactile feedback is often critical in vascular procedures but not supported by most widely available robotic systems. Finally, specialized training is required, since integrated vascular surgery trainees, unlike general surgery residents, have less early exposure to robotics, leading to a steeper learning curve and the need for supplementary training [24,25,26].

### 1.3. Purpose and Scope of the Review

The present review provides a comprehensive synthesis of the current role and future outlook of robotics in vascular surgery. It examines the technological platforms currently available, the spectrum of clinical applications across aortic, visceral, venous, and endovascular interventions, and the comparative advantages over traditional methods. Barriers to adoption, including economic, technical, and educational challenges, are critically appraised. Finally, we discuss emerging technologies such as artificial intelligence, augmented reality, microsurgical robotics, and telesurgery, and their potential to redefine vascular practice.

By integrating existing evidence with forward-looking analysis, this review aims to inform vascular surgeons and trainees about the opportunities and challenges inherent to this “third revolution” in vascular surgery, while also raising awareness of the key industry entities involved in an impartial manner.

## 2. Robotic Platforms and Technology

Over the last two decades, robotic surgery has matured from a niche innovation to an integral part of multiple surgical disciplines. In vascular surgery, the most widely adopted system remains the da Vinci Surgical System (Intuitive Surgical, Sunnyvale, CA, USA), which has undergone continuous refinements since its initial FDA clearance in 1997 for visualization and tissue retraction, and its subsequent approval for general surgical applications in 2000 [27]. As of the end of 2023, more than 9100 da Vinci systems are installed worldwide, spanning specialties such as urology, gynecology, colorectal, general, thoracic, and head and neck surgery [28]. Many vascular procedures performed on this platform are “off-label,” requiring strict adherence to institutional review board (IRB) protocols and informed consent procedures.

### 2.1. Core Components and Capabilities

The da Vinci system comprises three main elements: (i) a surgeon’s console providing high-definition stereoscopic (3D) visualization and intuitive control; (ii) a patient-side cart with multiple interactive robotic arms; and (iii) a vision cart that houses imaging and processing hardware. Key features relevant to vascular surgery include EndoWrist™ instruments with seven degrees of freedom, acting as an extension of the surgeon’s arm and allowing more ergonomic posture than traditional laparoscopy, 3D high-definition optics with true depth perception (up to 4K), and tremor filtration with motion scaling to translate larger hand movements into fine micromotions for precision suturing and dissection. These capabilities can facilitate complex tasks such as aortic anastomosis and potentially reduce aortic clamp times, hence, improving surgical outcomes [29,30].

### 2.2. System Iterations and Relevance to Vascular Surgery

The da Vinci Xi (introduced 2014) offers thinner, longer arms, uniform trocar sizes (8 mm), and flexible camera placement from any arm, advantages in multi-quadrant vascular cases. Early institutional experience with Xi-assisted vascular procedures (*n* = 379) demonstrated a 96.6% robotic completion rate, 0.26% 30-day mortality, and 0.5% late prosthetic infection. The da Vinci SP (single port; FDA-cleared 2018) introduces three articulated instruments and a 3D camera through a single 25–28 mm incision, with early use in urology, head and neck, colorectal, and thoracic surgery, and investigational applications in select vascular procedures [31,32,33,34,35,36,37,38].

### 2.3. Emerging and Competitive Platforms

Beyond da Vinci, new entrants are poised to diversify the market and drive down costs: Medtronic Hugo™ RAS (modular, portable), CMR Surgical Versius^®^ Cambridge, UK (lightweight arms, open console), and Johnson & Johnson Ottava™ (table-integrated). These developments may lower barriers to adoption in vascular surgery, particularly in centers constrained by budget or operating room space [35,36].

## 3. Clinical Applications of Robotic-Assisted Vascular Surgery

Robotic assistance has expanded to include aortic reconstructions, delicate venous, visceral repairs, hybrid endoleak management, and minimally invasive decompression for compressive vascular syndromes. While many applications are still limited to high-volume specialty centers, the evidence base for feasibility and safety is steadily growing. Notably, many of these robotic vascular procedures are performed by non-vascular surgeons, highlighting the need for specialized robotic training in vascular surgery as robotic technologies increasingly become fundamental to surgical practices [24].

### 3.1. Aortic Surgery

The most extensive application of robotics in vascular surgery to date has been in the management of aortoiliac disease [39]. Aortic reconstruction is challenging due to the need to control high-pressure, often heavily calcified arteries. The construction of a durable vascular anastomosis is a highly technical task that must be completed within a limited ischemic window after aortic clamping and complicated by the lack of haptic feedback in most robotic systems. Nonetheless, several case series have confirmed the procedure’s safety. A summary table was added compiling operative time, blood loss, conversion rates, complications, mortality, and patency across major robotic vascular series (Colvard, Desgranges, Garrett, Kolvenbach, Lin, Novotný, Rouby, Stadler, Sutter, Thiney, Diks) (Supplemental Table S1).

#### 3.1.1. Aortoiliac Occlusive Disease (AIOD)

The earliest robotic applications in vascular surgery were for aorto-bifemoral bypass in AIOD. Wisselink et al. first reported a robot-assisted laparoscopic case in 2002, and Martinez et al. later described the first fully robotic aorto-bifemoral bypass in 2009 [23,40]. Since then, multiple series, now totaling over 500 patients, have demonstrated reproducible outcomes. SSutter et al. evaluated 70 patients with TASC C and D aortoiliac lesions and found 48-month primary and secondary patency rates of 92% and 98.1%, respectively; by comparison, endovascular AIOD treatment yielded 5-year primary and secondary patency rates of 70% and 77%, respectively, while open aortobifemoral bypass demonstrated a 5-year primary and secondary patency rate of 86.2% and 96.5%, respectively [41]. A 2025 meta-analysis (595 patients) found a pooled conversion rate of 5% and a 30-day mortality of 3% for aortoiliac robotic procedures [40]. Advantages include ergonomic ease in constructing proximal aortic anastomoses, a traditionally challenging step in pure laparoscopy. The technique has also been applied to aorto-unifemoral and iliofemoral bypasses (Table 1) [42].

#### 3.1.2. Abdominal Aortic Aneurysm (AAA)

Robotic AAA repair remains technically demanding, requiring aneurysm sac management, lumbar artery control, and thrombus removal. In a 61-patient series, the conversion-to-open rate was 13.1% with a 30-day mortality of 1.6% [42]. Compared with AIOD repairs, AAA cases had significantly longer clamp times (95 min vs. 68 min; *p* = 0.006) and greater blood loss (1900 mL vs. 583 mL; *p* = 0.0002) [43], though conversion rates did not differ significantly (Table 2).

#### 3.1.3. Thoracic and Thoracoabdominal Aorta

Experience is limited but growing. Fernandez et al. described a robotic descending aorto-bifemoral bypass in a patient with hostile abdomen post-occluded graft, with aortic clamp time of 40 min [44]. Cadaveric feasibility studies suggest descending thoracic aorta replacement can be achieved robotically with preparation and clamp times of 64 and 44 min, respectively [45]. However, these early reports are limited by small sample size and lack of clinical follow up and therefore cannot provide robust data on outcomes such as durability, perioperative morbidity, or cost.

### 3.2. Visceral Artery Disease

#### 3.2.1. Splenic and Renal Artery Aneurysms (SAA, RAA)

The Society for Vascular Surgery recommends repair for symptomatic SAAs, those >3 cm, or in women of childbearing age; similar criteria apply to RAAs, with refractory hypertension as an additional indication [46]. Robotic repair enables spleen-preserving techniques and precise arterial reconstructions which is more challenging in conventional laparoscopy. A systematic review (53 patients) reported 0% perioperative mortality, 5.6% conversion, and 3.6% reintervention rates [47]. For RAAs, a 2024 review of 23 patients reported 100% technical success, warm ischemia times 19–82 min, and hospital stays 1–7 days (Table 3) [48].

#### 3.2.2. Hepatic Artery Aneurysms

Salloum et al. reported a successful robotic repair of a common hepatic artery aneurysm with aberrant anatomy, completed in 182 min with discharge on postoperative day five [49].

### 3.3. Venous Disease

#### 3.3.1. Inferior Vena Cava (IVC) Reconstruction

Large series (39 patients) have demonstrated robotic safety in complex IVC procedures, including tumor thrombus resection, filter removal, and tumor-invaded IVC resection. Median blood loss was 550 mL, with median clamp time 23 min [50]. Innovations include robotic thrombectomy using modified “tourniquet downstairs” techniques to reduce hepatic warm ischemia [51,52].

#### 3.3.2. Nutcracker Syndrome

**Figure 2:**
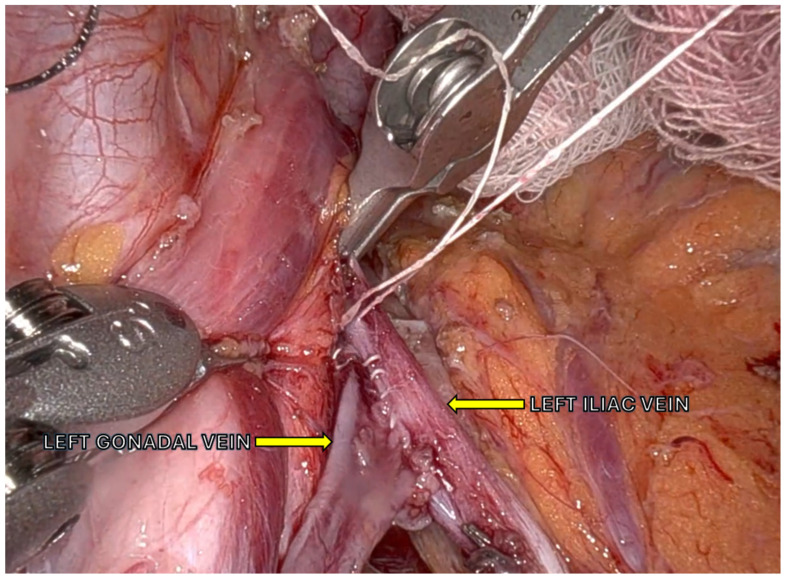
Robotic transposition of the left gonadal vein to the left common iliac vein.

Robotic left renal vein transposition has shown 100% patency and universal symptom improvement in a prospective series of 11 patients, though one case required thrombectomy and stenting for graft thrombosis [53,54,55]. Novel extravascular PTFE cuff placement has been described for minimally invasive decompression with symptom resolution (Figure 2) [56].

### 3.4. Type II Endoleak Management

**Figure 3:**
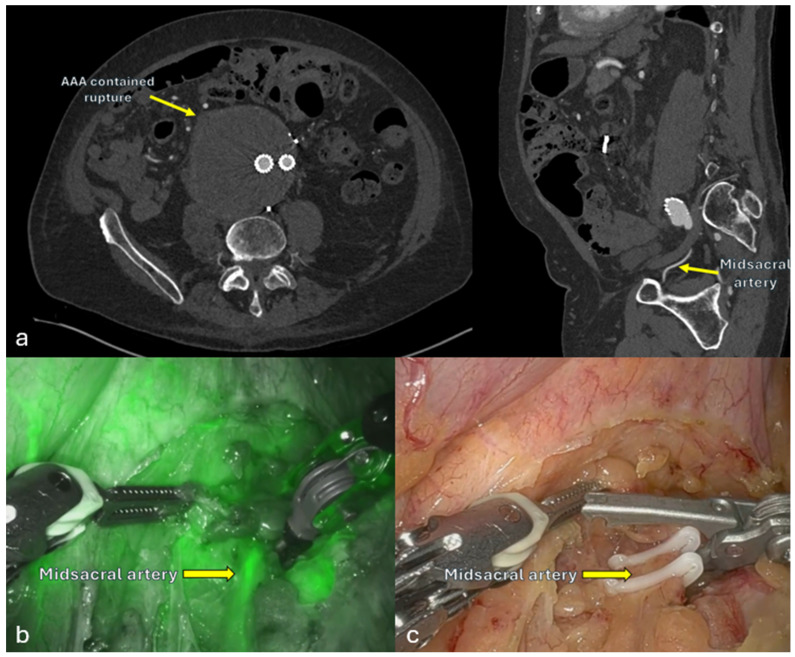
Robotic ligation of the midsacral artery for treatment of a type II endoleak. (**a**), Axial and sagittal CTA images demonstrating the abdominal aortic aneurysm. (**b**), Indocyanine green (ICG) fluorescence was used to identify the midsacral artery. (**c**), Robotic clipping of the identified artery.

For persistent type II endoleaks post-EVAR, robotic ligation of feeding vessels (e.g., inferior mesenteric or lumbar arteries) offers a minimally invasive alternative when endovascular therapy fails. Reported cases show high technical success and minimal morbidity (Figure 3) [57,58]. While experience with robotic endoleak management is still limited, a systematic review of laparoscopic inferior mesenteric artery ligation for type II endoleaks reported a 100% technical success rate, 0% mortality, and a 15.1% reintervention rate.

### 3.5. Median Arcuate Ligament Syndrome (MALS)

**Figure 4:**
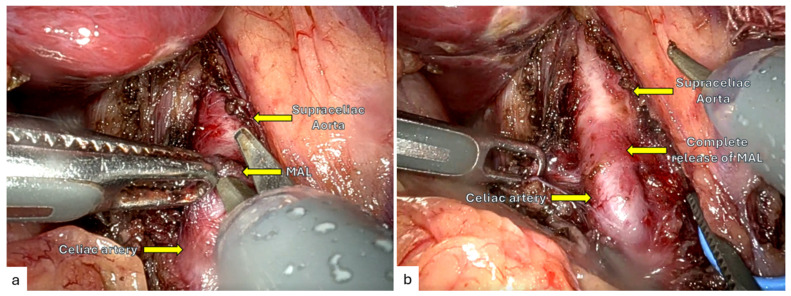
(**a**), Robotic release of the median arcuate ligament (MAL). (**b**), Complete release of the MAL.

Robotics is well-suited to the delicate periaortic dissection required for MALS decompression. A 2024 systematic review (290 patients) found mean operative time 117 min, estimated blood loss 5–30 mL, and conversion rate 1.37%, with hospital stays < 2 days (Figure 4) [59,60,61,62,63,64].

### 3.6. Thoracic Outlet Syndrome (TOS)

TOS comprises disorders from neurovascular bundle compression (brachial plexus, subclavian vessels) at the thoracic outlet [65]. Conventional open approaches (transaxillary, supraclavicular, and infraclavicular) are efficacious but limited by visualization constraints and risks to adjacent structures like the brachial plexus or phrenic nerve. Robotic transthoracic techniques mitigate these via enhanced 3D magnification, involving intercostal ports, CO_2_ insufflation, scalene division, first rib resection, and neurolysis [66,67]. A 2023 systematic review of 12 studies and 397 patients reported mean operative time of 133 min, blood loss of 79.5 mL, hospital stay of 2.57 days, and symptom resolution in 91–100% of cases [68].

### 3.7. Aberrant Subclavian Artery (ASA) Repair

Aberrant subclavian artery (ASA), or arteria lusoria, is a congenital aortic arch variant potentially causing dysphagia lusoria, especially with Kommerell’s diverticulum. A hybrid robot-assisted approach, thoracoscopic ASA ligation plus open supraclavicular bypass, serves as a minimally invasive option to thoracotomy [69]. In a nine-patient series, mean operative time was 169 min, with no transfusions, hospital stay of 2 days, and dysphagia improvement in most (significant in five, moderate in one, mild in one; one unchanged, one lost to follow-up); however, the reported nine-patient series should be regarded as preliminary evidence rather than definitive proof of safety or efficacy [70].

## 4. Advantages and Benefits

### 4.1. Patient-Centered Benefits

Robotic access reduces surgical trauma by avoiding large incisions, resulting in less blood loss, less postoperative pain, improved cosmesis, and shorter hospitalization [21,25]. Patients typically resume oral intake and ambulation sooner than after open repair. Minimally invasive access also reduces laparotomy-related complications, including incisional hernias and postoperative bowel obstruction. In a meta-analysis of robotic coronary artery bypass (RCAB) versus conventional CABG, pneumonia (OR 0.09, 95% CI 0.01–0.75; *p* = 0.03) and wound infection (OR 0.17, 95% CI 0.03–0.90; *p* = 0.04) were significantly lower in the robotic cohort [71]. Stedler et al. evaluated 298 patients undergoing robot-assisted aortic procedures and reported significantly reduced blood loss compared with laparoscopy (571 mL vs. 1680 mL) and shorter clamp times (204 min vs. 290 min). While some benefits are extrapolated from other specialties, these findings highlight potential improvements in vascular outcomes.

### 4.2. Surgeon-Centered Benefits

High-definition 3D optics, motion scaling, and tremor filtration enable precise vascular dissection and anastomosis, particularly in deep or confined spaces [72,73,74]. Console ergonomics reduce musculoskeletal strain relative to open and conventional laparoscopic surgery, potentially decreasing work-related injuries [75]. Compared with laparoscopy, robotic suturing and knot-tying; benefiting from wristed instrumentation and 3D visualization, facilitating skill acquisition in a similar number of cases, which is critical for complex vascular anastomoses. Lucereau et al. conducted a randomized trial comparing the learning curves for laparoscopic (Group A) versus robotic (Group B) aortic anastomoses over ten cases. In Group A, anastomosis time decreased from 2340 ± 64 s on the first case to 651 ± 248 s on the tenth (*p* < 0.05). In Group B, time decreased from 1989 ± 556 s to 801 ± 120 s (*p* < 0.05). The initial anastomosis was significantly faster in the robotic group (*p* < 0.05), while times on the tenth case did not differ significantly between groups [22,25].

## 5. Challenges and Barriers to Widespread Adoption

Despite the demonstrated feasibility and benefits in selected applications, the adoption of robotics in vascular surgery has been significantly slower than in other specialties like urology or gynecology. Several substantial challenges related to economics, training, technology, and clinical evidence remain, creating a high barrier to entry for many programs.

### 5.1. Economic Constraints

Robotic systems require substantial capital investment (USD 1.5–2.0 million) and annual maintenance (USD 100,000–180,000) [25]. Instruments are limited-use or single-use, generating recurring costs. The overall cost-effectiveness compared to open or endovascular repair is still under debate and requires more rigorous, long-term analysis to account for potential savings from shorter hospital stays and fewer complications. Vascular-specific cost-effectiveness data remains scarce, and most published analyses are extrapolated from other surgical specialties.

Sanmartin et al. evaluated the cost-effectiveness of remote robotic thrombectomy. Using a U.S. Markov model, robotic thrombectomy was less costly (USD 321,269 vs. 321,397) and more effective (4.05 vs. 3.88 QALYs) than the standard transfer strategy, remaining cost-effective in 90% of simulations at a $100,000/QALY threshold. These findings suggest that although robotic platforms require substantial upfront investment, they may reduce disability-related costs and generate quality-adjusted life-year gains.

### 5.2. Training and Learning Curve

Developing and maintaining proficiency in robotic vascular surgery presents a formidable educational challenge.

Steep Learning Curve for Complex Procedures: While robotic suturing may be easier to learn than its laparoscopic counterpart, mastering a complex procedure such as a robotic aortobifemoral bypass still involves a significant learning curve. In a prospective series of 70 patients with Trans-Atlantic Inter-Society Consensus C and D lesions, the conversion-to-open rate stabilized at 10% after the first 13 cases, indicating early proficiency in safety. Operative efficiency, measured by mean procedure duration, plateaued at approximately 220 min only after 35 cases [41].

Lack of Standardized Training and Credentialing: There is no universally accepted, validated training pathway for vascular surgeons to become proficient in robotic surgery. Most vascular surgeons lack foundational experience in advanced laparoscopy, making the transition more difficult. The development of a structured, simulation-based curriculum is considered essential but is not yet widely implemented. European Society for Vascular Surgery has used Delphi surveys to identify core vascular procedures ideal for simulation-based education, but access to and integration of this training remains a challenge. Simulation models tailored to vascular procedures have also been trialed. For example, Gamberini et al. described a high-fidelity vascular simulator embedding soft strain sensors into vessel analogs, enabling real-time feedback on vessel handling forces. The system demonstrated face, content, and construct validity by reliably disguising novices from experts, underscoring the potential for simulation-based assessment and training in robotic vascular surgery. However, such vascular-specific simulators remain in early adoption stages and are not yet standardized or widely available. Lengyel et al. proposed a structured pathway beginning with basic robotic skills, manufacturer-provided simulation, and wet labs, followed by supervised clinical cases of increasing complexity. Štádler et al. emphasized the need for prior laparoscopic experience, simulator training, animal/cadaver labs, and dual-console mentorship. Credentialing frameworks should be procedure-specific, requiring supervised cases, proof of proficiency, and maintenance through annual volumes or validated simulation. Institutional commitment, academic support, and society-level guidelines will be essential for broader adoption.

Recommendations for Training and Credentialing: To ensure safe adoption, we recommend a standardized, stepwise training pathway: (i) credentialing in the equivalent open/endovascular procedure; (ii) modular curricula beginning with manufacturer-provided simulation, progressing to vascular-specific models, cadaveric labs, and dual-console training; and (iii) supervised cases of escalating complexity. Hospitals should grant procedure-specific robotic privileges with minimum case requirements and ongoing proficiency verified by annual case volume or validated simulation. Fellowship curricula and continuing education for practicing surgeons should integrate robotics. Professional societies should establish consensus guidelines, promote validated simulators, and support multicenter registries to track outcomes and training benchmarks.

Need for a Dedicated Team: A successful robotic program requires a dedicated and highly proficient team, including the console surgeon, bedside assistant, scrub technicians, and anesthesiologists. Inconsistent team members can negatively impact efficiency and safety, especially during the challenging learning curve phase.

### 5.3. Technological Limitations

Current robotic systems have inherent limitations that are particularly relevant to the unique demands of vascular surgery.

Lack of Haptic (Tactile) Feedback: The absence of tactile feedback is a primary drawback of most currently available robotic systems. Surgeons cannot “feel” the tissue and must rely entirely on visual cues to gauge tissue tension and suture traction, which can lead to inadvertent tissue damage or suture breakage. The clinical risk of this limitation was starkly highlighted in a case report where undetected mechanical compression by a robotic arm led to an external iliac artery dissection and acute limb ischemia [76]. While newer systems like the da Vinci 5 and HUGO™RAS are beginning to incorporate force feedback, this technology is not yet widespread [27,72,77,78].

Large Footprint and Cumbersome Setup: The physical size of the patient-side cart can be cumbersome in the operating room, limiting the bedside assistant’s access to the surgical table and potentially leading to external conflicts between the robotic arms. The processes of docking and undocking the robot can also add to the overall procedure time [79].

Challenges of Emergency Conversion: Converting to an open procedure during a vascular emergency is complex and fraught with unique challenges. Safety mechanisms in newer robots can lock instruments in place if the camera is removed from the patient, creating a critical and potentially catastrophic delay. A case report detailed an aortic injury that was exacerbated because the robotic grasper could not be disengaged after an emergency laparotomy was performed [76]. A survey of robotic team members found that 34% did not recall receiving training in emergency undocking procedures, highlighting a critical safety knowledge gap [76,80,81]. Developing and adopting standardized emergency protocols may help address these gaps. For example, one of the most validated approaches is the Robotic Undocking for Life Emergency Support (RULES) protocol. This standardized training program demonstrated significant improvements in team performance, reducing undocking time by 66% in trained teams compared to only 20% improvement in control groups. The protocol emphasizes specific actions and commands for safe emergency robotic undocking while developing both technical and non-technical skills across surgical team members.

### 5.4. Limited High-Level Evidence

Most published data are single-center, retrospective series. A 2025 meta-analysis judged all 10 observational studies to carry serious risk of bias (ROBINS-I), mainly due to confounding and selection bias [43]. Multicenter randomized controlled trials comparing robotic, open, and endovascular techniques are needed to define clinical effectiveness and cost-effectiveness.

### 5.5. Lack of Dedicated Vascular Instruments

A critical and frequently cited barrier is the lack of a full suite of robotic instruments designed specifically for the needs of vascular surgery.

Absence of Dedicated Vascular Clamps: Surgeons currently rely on laparoscopic clamps introduced through separate assistant ports or on techniques like the Rummel tourniquet for vascular control. There are no specialty-focused robotic aortic clamps that can be controlled from the surgeon’s console, a significant limitation for a field that is fundamentally centered on vascular control.

Instrument Handling Issues: The powerful grip of robotic needle drivers, combined with the lack of haptic feedback, makes them prone to breaking delicate monofilament sutures like Prolene. This often necessitates the use of more durable but potentially less ideal materials like PTFE sutures for vascular anastomoses [24].

## 6. Robotic Applications in the Endovascular Realm

The application of robotics in the endovascular domain represents a distinct and rapidly evolving frontier. Endovascular robotics seeks to enhance the minimally invasive catheter-based procedures that define modern vascular intervention. The rationale for this technology stems from the inherent challenges of manual endovascular surgery. Vascular navigation is a demanding skill that requires the operator to translate 2D fluoroscopic images into a 3D mental map, all while manipulating long, flexible instruments through a delicate and often tortuous vascular tree. The surgeon must rely on limited visual information and subtle haptic cues such as the small axial forces and torques sensed at their fingertips to guide the instruments and avoid unintentional contact with vessel walls, which carries the potential for perforation, dissection, and embolic events. Furthermore, this reliance on 2D fluoroscopy exposes both the patient and the medical team to ionizing radiation and requires the use of potentially nephrotoxic contrast agents. Endovascular robotics aims to address these limitations by introducing greater precision, stability, and operator safety.

### 6.1. Rationale and Advantages

The primary advantages of integrating robotics into endovascular procedures are centered on enhancing surgeon capability and improving the safety profile for both the patient and the clinical team.

Enhanced Precision and Stability: Robotic platforms can translate a surgeon’s commands into movements with a precision that is humanly impossible. These systems allow for sub-millimeter advancements and retractions of catheters and guidewires, along with fine rotational control. This eliminates the physiological hand tremor that can be a factor in manual manipulation. A key benefit is stability; once a position is achieved, the robotic arm can hold an instrument motionless, which is invaluable during sensitive tasks like stent deployment or balloon inflation. Clinical studies have shown this stability leads to less catheter-to-wall contact, which in turn reduces the number of cerebral microemboli (High-Intensity Transient Signals, or HITS) during navigation in the aortic arch, suggesting a potential for improved procedural safety [82,83,84,85,86,87,88,89,90,91]. Another study reported that the use of robotic endovascular catheters in transcatheter aortic valve implantation reduces contact with the aortic arch wall in compared to the manual techniques (1 (0–5) vs. 6 (2–22)), potentially reducing the embolic risk during endovascular manipulation.

Significant Reduction in Operator Radiation Exposure: Perhaps the most significant and well-documented advantage is the dramatic reduction in radiation exposure for the operator. In a standard endovascular procedure, the surgeon stands next to the patient, exposed to scatter radiation from the C-arm. Robotic systems are designed in a teleoperated configuration, allowing the surgeon to sit at a remote, radiation-shielded workstation or “cockpit” to control the devices. Endovascular robotics enhances catheter navigation precision and reduces operator radiation exposure by 85–95%, with multiple series demonstrating consistent benefit compared to manual techniques. This drastically mitigates the long-term occupational health risks associated with chronic radiation exposure, including an increased incidence of cancers, cataracts, and orthopedic injuries from the strain of wearing heavy lead aprons [92,93,94,95,96,97,98,99].

Fluoroscopy Time and Contrast Utilization: Costa et al. compared fluoroscopy time and contrast usage in robotically assisted versus manual neuro-endovascular procedures found similar fluoroscopy times (median 12 vs. 12 min, *p* = 0.69), and lower contrast volumes in the robotic group, although the difference did not reach statistical significance (82 vs. 92 mL, *p* = 0.54).

### 6.2. Platforms: Past and Present

The endovascular robotics market has seen a variety of platforms developed by different companies, each with unique technologies and varying degrees of commercial success.

Hansen Medical (Auris Health Inc. Redwood City, CA, USA)/Johnson & Johnson MedTech (New Brunswick, NJ, USA): This company pioneered the Magellan and Sensei X2 systems. These platforms were based on electromechanical, tendon-driven (pull-wire) steerable catheters and were used in both peripheral endovascular and cardiac electrophysiology (EP) procedures. Although they demonstrated the feasibility of robotic navigation, both systems are no longer commercially available after Hansen Medical was acquired by Auris Health, which was later incorporated into Johnson & Johnson [84,100,101,102,103,104].

Stereotaxis (St. Louis, MO, USA): This company’s Niobe and newer Genesis systems utilize a different approach based on magnetic navigation. These platforms, used primarily for cardiac EP, employ two large, externally positioned permanent magnets to generate a magnetic field that precisely controls the tip of a magnetically enabled catheter. This allows for very soft, atraumatic navigation and is often integrated with 3D anatomical mapping systems like CARTO 3 (Biosense Webster, Diamond Bar, CA, USA) [105,106].

Corindus (Siemens Healthineers (Enlargen, Germany)): The CorPath 200 and its successor, the CorPath GRX, are electromechanical systems designed to mechanically drive standard interventional instruments under fluoroscopic guidance. While initially developed for percutaneous coronary intervention (PCI), the GRX system received clearance for peripheral and, more recently, neurovascular interventions. However, in May 2023, Siemens Healthineers announced it would discontinue the platform for cardiac applications due to an unfavorable cost–benefit ratio and lower-than-expected adoption, shifting its focus exclusively to the promising neurovascular market [84,107,108,109,110].

### 6.3. Emerging Platforms

The field continues to evolve with new systems designed to address the limitations of earlier platforms.

Robocath R-One+ and Catheter Precision Amigo RCS target compatibility with standard devices for workflow and cost advantages. Flux One/Nanoflex (magnetic navigation), Sentante (haptic feedback), and the Liberty Robotic System (disposable architecture) represent active development paths [1,111,112,113,114,115,116].

### 6.4. Current Applications

The clinical use of endovascular robotics has been explored across a variety of vascular domains, with feasibility and safety demonstrated in several key areas.

Peripheral Artery Disease (PAD): The Robotic peripheral vascular intervention (RAPID trials) demonstrated the safety and feasibility of the CorPath system for robotically assisted peripheral vascular intervention in the femoropopliteal segment, successfully performing balloon angioplasty and stenting [107].

Carotid Artery Stenting (CAS): Several studies have reported the successful use of robotic systems for CAS. The enhanced stability and precision are particularly valuable when navigating the aortic arch and placing stents in the carotid artery, a procedure where precision is critical to prevent neurologic complications. These studies report high technical success rates (often 100%) and a rapid learning curve. However, the available studies are limited to small cohorts and primarily demonstrate feasibility and safety [117,118].

EVAR: Robotics has been applied to assist with the most challenging steps of complex aortic repair. The Magellan system was used successfully to facilitate target vessel cannulation during fenestrated endovascular aneurysm repair (FEVAR), providing a stable platform for this difficult maneuver. More recently, the novel ALLVAS robot demonstrated the ability to perform an entire standard EVAR procedure from start to finish [119,120]. However, Robotic EVAR experience remains largely confined to highly specialized centers with dedicated expertise, and reproducibility across broader practice settings has yet to be demonstrated.

Visceral and Neurovascular Interventions: The application is expanding to smaller, more tortuous vessels. Case reports have described the successful robot-assisted coil embolization of hepatic and renal artery aneurysms. The neurovascular space has become a major focus, with systems being evaluated for the treatment of intracranial aneurysms [47,121,122,123].

## 7. Future Perspectives and Innovations

Rapid advancements in robotics, artificial intelligence, and imaging are poised to transform vascular surgery, overcoming current limitations and expanding applications into increasingly complex domains, potentially establishing robotics as the third major paradigm in vascular patient care.

### 7.1. Technological Advancements on the Horizon

Future iterations of robotic surgery will feature more intelligent, compact, and versatile systems that integrate seamlessly with sophisticated imaging and data analytics, fostering a more intuitive and secure operative setting (Table 4).

#### 7.1.1. Integration with Artificial Intelligence (AI)

AI is poised to transform every phase of the surgical journey. The fusion of AI with robotic systems can enhance surgical decision-making and automate routine tasks.

Preoperative Planning and Simulation: AI algorithms can analyze preoperative imaging (CT, MRI) to create patient-specific 3D anatomical models that are highly accurate and re-producible. These reconstructions allow surgeons not only to visualize vascular anatomy but also to identify subtle anatomical variants, measure vessel diameters, and assess spatial relationships between target vessels and adjacent structures. Incorporating these models into virtual or augmented reality environments enables surgeons to rehearse complex procedures step by step, simulate catheter trajectories, and test different device configurations before entering the operating room. Recent studies demonstrate that AI-driven segmentation and modeling significantly reduce the time needed for 3D reconstruction compared with manual methods, making patient-specific rehearsal more feasible in routine clinical practice. Furthermore, AI-enabled simulations can be used to predict procedural challenges, such as difficult cannulation angles or risk of endoleak, and can help optimize strategy selection, thereby improving preparedness and potentially reducing intraoperative errors and operative time [27,124,125,126].

Intraoperative Guidance: Machine learning models can provide real-time intraoperative guidance. One of the key limitations of augmented reality (AR) in surgery is that overlaid 3D anatomical models can obscure instruments, impairing visibility and depth perception. A first-in-human study addressed this by deploying a deep-learning pipeline that automatically segments surgical tools and ensures they remain visible within the AR field. Running at 80 fps with <15 ms latency, the system restored depth perception and tool visibility during robotic partial nephrectomy, endovascular stent retrieval, and liver metastasectomy, where surgeons reported improved navigation, tumor localization, and confidence in intraoperative decision-making [127,128,129].

Automation of Surgical Sub-Tasks: Ongoing research aims to empower robots to independently execute repetitive and straightforward tasks, such as suturing, knot-tying, and complete anastomoses, under surgeon oversight. This approach may alleviate surgeon fatigue while boosting consistency and efficiency in these essential steps [130,131,132,133].

#### 7.1.2. Next-Generation Robotics

The hardware of surgical robotics is evolving to become more specialized, accessible, and capable, moving beyond the one-size-fits-all model.

Smaller, More Affordable Systems: The introduction of new platforms from competitors like Medtronic (Hugo), CMR Surgical (Versius), and Johnson & Johnson (Ottava) is expected to drive down costs and encourage the development of smaller, more specialized robotic systems tailored to specific procedures, making the technology more accessible [134,135].

Enhanced Haptic Feedback: A major focus of development is the integration of true haptic feedback. The newest da Vinci 5 system incorporates force feedback, and ongoing research into advanced tactile sensors and haptic interfaces promises to fully restore the surgeon’s sense of touch. This could dramatically improve safety, especially during delicate dissection and suturing, and reduce the risk of tissue damage or suture breakage [77].

Microsurgery Platforms: Specialized robotic systems, such as the Symani Surgical System, are being developed specifically for microsurgery. These platforms, with their extreme precision and tremor elimination, are ideal for creating microvascular anastomoses (e.g., in lymphatic reconstruction) and hold immense potential for complex distal vessel repair in vascular surgery [136].

#### 7.1.3. Augmented Reality (AR) and Virtual Fixtures

AR technology allows for the real-time fusion of virtual information with the surgeon’s view of the operative field, creating an information-rich surgical environment.

Image Overlay for Precise Navigation: AR systems can project 3D vascular reconstructions from preoperative CT or MRI scans onto the patient’s anatomy or the surgical display. This provides a “see-through” capability, guiding surgeons to target vessels for access or dissection with greater accuracy and potentially reducing radiation and contrast use in endovascular procedures. Head-mounted displays (HMDs) like the Microsoft HoloLens are being explored to provide an ergonomic, hands-free visualization platform [129,137].

Virtual Fixtures and “No-Go” Zones: Software can create “virtual fixtures” or software-defined “no-go” zones around critical structures like nerves or major vessels. The robotic system would then provide resistance or prevent the instruments from entering these zones, adding a crucial layer of active safety to prevent inadvertent injury [138].

#### 7.1.4. Soft Robotics and Steerable Catheters

Inspired by biological organisms, soft robotics utilizes flexible and compliant materials to create adaptable instruments capable of navigating delicate and complex environments with minimal trauma.

Flexible and Adaptable Instruments: Soft robotic catheters and instruments can bend and conform to the tortuous and fragile anatomy of the vascular system, reducing the risk of vessel trauma during navigation. This technology is particularly promising for endovascular applications, where it could enable safer access to challenging distal vessels [139,140].

Biomimetic Designs: Research into biomimetic robotic systems, which mimic biological principles, aims to create more intuitive and effective surgical tools. This includes the development of catheters with enhanced steerability and instruments with novel gripping mechanisms inspired by natural forms, moving away from rigid metallic designs [141,142].

### 7.2. Expanding the Clinical Frontier

These technological advancements are set to expand the scope of robotic vascular surgery into more complex and previously inaccessible areas, offering novel solutions to longstanding clinical problems.

Complex minimal invasive Hybrid Procedures: The combination of robotic and endovascular techniques in a single hybrid setting is a promising frontier. For instance, a surgeon could perform a complex robotic debranching of visceral arteries to prepare a landing zone for a subsequent endovascular stent graft, all within the same procedure. This approach could potentially reduce overall procedural cost and morbidity for complex aortic pathologies, as it avoids the need for a full open repair while leveraging the conventional surgical durability.

Telesurgery: Enabled by high-speed 5G networks and advanced robotic platforms, telesurgery could allow experienced surgeons to mentor or even perform procedures on patients in different geographic locations. This technology has the potential to dramatically expand access to expert care, allowing specialists in high-volume centers to treat patients in rural or underserved areas [143]. The Toumai robot has already demonstrated 5G-enabled remote surgical capabilities over a distance of ~5000km. The ultimate application of this technology could even extend to providing surgical care in extreme remote environments, such as war zones and space [144].

Difficult Rescue and Reduced Radiation: Robotic systems, particularly for endovascular procedures, can facilitate “difficult rescue” scenarios where conventional access is challenging. The enhanced stability and precision of robotic catheters can help navigate tortuous anatomy or cannulate challenging vessel origins, potentially with less radiation exposure than multiple failed manual attempts [84].

### 7.3. Other Challenges Facing AI Adoption

Beyond technological innovation, the widespread adoption of next-generation robotic vascular surgery will be constrained by significant ethical and regulatory challenges.

#### 7.3.1. Ethical Challenges

Incorporating artificial intelligence (AI) into medicine presents significant ethical challenges that must be addressed to ensure safe, fair, and trustworthy deployment. Key concerns include maintaining patient autonomy through transparent informed consent, where patients understand the role, limitations, and decision-making processes of AI systems. Data privacy and security are critical, as AI relies on large datasets containing sensitive health information, requiring strict compliance with regulations such as HIPAA and GDPR. There is also the risk of bias in AI algorithms if training datasets lack diversity, potentially leading to unequal care outcomes. Accountability for errors remains legally unclear, raising questions about whether responsibility lies with the surgeon, the device manufacturer, or the algorithm developer. Furthermore, ensuring equitable access to AI-enabled healthcare is essential, as advanced technologies risk being concentrated in high-resource centers, potentially exacerbating disparities in healthcare delivery. Ethical integration of AI thus demands robust governance frameworks, transparency, fairness, and policies that address both patient safety and social equity.

#### 7.3.2. Regulatory Challenges

AI regulation varies globally, shaped by distinct national policies and legal frameworks. In the European Union, the 2024 AI Act provides a comprehensive legal structure governing AI development and deployment, defining responsibilities for developers and distributors, regulating high-risk applications, and outlining safeguards for prohibited uses. In the United States, a 2023 executive order established principles for the safe and trustworthy development of AI, addressing definitions, testing, privacy, and security, although its full implementation remains in progress. The U.S. Food and Drug Administration (FDA) classifies AI and machine learning tools as software as a medical device (SaMD) and has created a digital health advisory committee to guide their regulation. Despite approving numerous AI/ML tools, the regulatory landscape continues to evolve in response to technological advances and emerging insights into AI’s benefits and risks.

### 7.4. The Path Forward

For robotic vascular surgery to move from a niche application to a mainstream modality, a concerted effort across several fronts is required from the entire vascular community.

Building a Strong Evidence Base: The most critical need is for high-quality clinical evidence. This requires a shift from small case series to large, prospective registries and, ideally, multicenter randomized controlled trials comparing robotic techniques to established open and endovascular procedures. Such studies are essential to definitively establish the safety, efficacy, and cost-effectiveness of robotic approaches.

Standardizing Training and Education: A clear, standardized training pathway for vascular trainees and practicing surgeons is paramount [145,146,147,148,149,150]. This pathway must incorporate:

- Simulation-Based Training: Proficiency-based simulation is essential for developing foundational robotic skills in a safe, controlled environment before transitioning to patient care. Needs assessments have identified core vascular procedures, such as anastomosis creation and endovascular navigation, as high-yield targets for inclusion in simulation-based curricula.
- Structured Fellowships and Proctoring: Advanced fellowships and structured proctoring by experienced robotic surgeons are necessary to guide surgeons through the steep learning curve of complex procedures.
- Collaboration and Innovation: Continued progress will depend on close collaboration between academic medical centers, surgical societies, and industry partners. This collaboration is needed to drive the development of dedicated robotic vascular instruments (e.g., robotic clamps, sutureless devices), refine surgical techniques, and support the clinical trials necessary to validate the technology and secure regulatory approvals.

## 8. Review Limitations

The current evidence base is limited. Most cited studies are observational and retrospective, often from high-volume centers, which may limit generalizability. Higher-level evidence such as randomized controlled trials and large-scale prospective registries are lacking. These gaps underscore the need for robust studies to validate safety, efficacy, cost-effectiveness, and long-term durability.

## 9. Conclusions

Robotic technology is an emerging frontier in vascular surgery, offering the potential to combine the durability of open repair with the benefits of minimally invasive techniques. Early experience in complex aortoiliac, visceral, and endovascular procedures demonstrates feasibility, safety, and enhanced precision, while reducing radiation exposure to surgeons.

However, high costs, limited vascular-specific instrumentation, lack of tactile feedback, and steep learning curves remain barriers to adoption. Standardized, simulation-based training and robust credentialing pathways are essential, as is strengthening the evidence base with multicenter randomized trials.

Future integration with artificial intelligence, augmented reality, and next-generation robotic platforms could enable more precise, less invasive, and widely accessible vascular interventions, including telesurgery. With focused collaboration, rigorous training, and strong evidence generation, robotics can become the next paradigm shift in vascular care.

## Figures and Tables

**Table 1:** Comparison of Outcomes for AIOD Treatment.

	Robotic	Open	Endovascular
Primary patency	92%	86.2%	70%
Secondary patency	98.1%	96.5%	77%
30-day mortality	3%	3.6% [3]	1.3% [4]

**Table 2:** Comparison of Outcomes for AAA Treatment.

	Robotic	Open	Endovascular
30-day mortality	1.6%	3.7% [5]	1.3% [5]

**Table 3:** Comparison of Outcomes (Reintervention and Mortality Rates) for SAA Treatment (Robotic vs. Open vs. Endovascular).

	Robotic	Open	Endovascular
Reintervention	3.6%	5.1% [6]	0.6% [6]
30-day mortality	0%	0.5% (per-year) [6]	3.2% (per-year) [6]

**Table 4:** Potential research agenda in robotic vascular surgery.

Priority Area	Research Needs/Examples	Expected Impact
Randomized control trial (RCT)	Robotic vs. open/endovascular repair for AAA, TOS, visceral aneurysms	- Establish comparative safety and efficacy
Registries	Robotic vascular surgery registry in the vascular quality initiative (VQI)	- Real-world outcomes - Complication tracking
Cost-effectiveness	Modeling QALYs, reimbursement strategies, hospital cost–benefit	- Support reimbursement and policy adoption
Technology development	Robotic platforms, advanced instruments, haptics	- Improve precision - Expand indications
Training and credentialing	Simulation validation studies, fellowship models, competency metrics	- Standardized training - Improve safety
Patient-centered outcomes	Quality of life questionnaires, pain scores, functional recovery, return to work	- Higher postoperative QoL scores - Quicker return to baseline activities - Capture the real-world impact and economic value

## Data Availability

No new data were created or analyzed in this study. All information synthesized in this review was obtained from previously published literature.

## Supplementary Materials

The following supporting information can be downloaded at: https://www.mdpi.com/article/10.3390/jcm14207353/s1, Table S1: Summary of clinical outcomes across the literature, AAA: abdominal aortic aneurysm, IA: Isolated Iliac Aneurysm, AIOD: Aortoiliac Occlusive Disease [22,25,41,151,152,153,154,155,156,157,158].
