# Supplementary material for: Robotic-Assisted Vascular Surgery: Current Landscape, Challenges, and Future Directions
Source: J Clin Med. 2025 Oct 17;14(20):7353. doi: 10.3390/jcm14207353 (PMC12565110; doi:10.3390/jcm14207353)
Supplement: Supplementary file 1 [file jcm-14-07353-s001.zip › jcm-3848170-supplementary.pdf]

Supplemental Table S1. Summary of clinical outcomes across the literature, AAA: abdominal aortic aneurysm, IA: Isolated Iliac Aneurysm, AIOD: Aortoiliac Occlusive Disease.

Study (Year)	N	Pathology	Operative Time (min)	Blood Loss (mL)	Conversion to Open	Complication	30-Day Mortality	Patency
Colvard et al. (2019) [151]	4	IA	397.6 ± 79.1	1594.9 ± 619.5	0.0	0.0	0.0	100% (6 months)
Desgranges et al. (2004) [152]	5	AIOD	NR	NR	20%	0.0	0.0	100% (6 weeks)
Garrett et al. (2008) [153]	6	AIOD	406.30 ± 64.3	1003.1 ± 663.2	16.7%	20%	0.0	NR
Kolvenbach et al. (2004) [154]	10	AAA	242.5 ± 40.5	NR	0.0	6.3	0.0	NR
Lin et al. (2011) [155]	19	AIOD (12) AAA (6) IA (1)	438.0 ± 92.0	1083.0 ± 581.0	4.8%	NR	0.0	NR
Novotný et al. (2011) [22]	40	AIOD	301.5 ± 68.4	333.3 ± 266.6	5%	2.3%	0.0	95% (48 months)
Rouby et al. (2019) [156]	46	AAA	NR	NR	8.7%	NR	0.0	97.7% (48 months)
Stadler et al. (2016) [25]	310	AAA (61) AIOD (224)	206.6 ± 36.5	632.1 ± 604.16	3.2%	3%	0.0	NR
Sutter et al. (2024) [41]	70	AIOD	218.7 ± 41.1	450.9 ± 404.4	4.3%	24.3%	1.4%	92% (48 months)
Thiney et al. (2019) [157]	85	AAA (24) AIOD (63)	NR	NR	3.5%	NR	4.7%	NR
Diks et al. (2007) [158]	17	AIOD	365 ± 91	1291.2± 1354.8	17.6%	NR	5.9%	100% (6 months)
